# Excessive milk production during breast-feeding prior to breast cancer diagnosis is associated with increased risk for early events

**DOI:** 10.1186/2193-1801-2-298

**Published:** 2013-07-03

**Authors:** Emma Gustbée, Charlotte Anesten, Andrea Markkula, Maria Simonsson, Carsten Rose, Christian Ingvar, Helena Jernström

**Affiliations:** Division of Oncology, Department of Clinical Sciences, Lund, Lund University, Barngatan 2B, Lund, SE-221 85 Sweden; CREATE Health and Department of Immunotechnology, Lund University, Medicon, Village, Building 406, Lund, Sweden; Division of Surgery, Department of Clinical Sciences, Lund, Lund University and Skane University Hospital, Lund, Sweden

**Keywords:** Breast cancer, Breast-feeding, Milk production, Early breast cancer events, Reproductive factor, Prognosis

## Abstract

Breast-feeding is a known protective factor against breast cancer. Breast-feeding duration is influenced by hormone levels, milk production, and lifestyle factors. The aims were to investigate how breast-feeding duration and milk production affected tumor characteristics and risk for early breast cancer events in primary breast cancer patients. Between 2002 and 2008, 634 breast cancer patients in Lund, Sweden, took part in an ongoing prospective cohort study. Data were extracted from questionnaires, pathology reports, and patients’ charts from 592 patients without preoperative treatment. Breast-feeding duration ≤12 months of the first child was associated with higher frequency of ER+/PgR+ tumors (P=0.02). Median follow-up time was 4.9 years. Higher risk for early events was observed for breast-feeding duration of first child >12 months (LogRank P=0.001), total breast-feeding duration >12 months (LogRank P=0.008), as well as ‘excessive milk production’ during breast-feeding of the first child (LogRank P=0.001). Patients with ‘almost no milk production’ had no events. In a multivariable model including both ‘excessive milk production’ and breast-feeding duration of the first child >12 months, both were associated with a two-fold risk for early events, adjusted HRs 2.33 (95% CI: 1.25-4.36) and 2.39 (0.97-5.85), respectively, while total breast-feeding duration was not. ‘Excessive milk production’ was associated with a two-fold risk of early distant metastases, adjusted HR 2.59 (1.13-5.94), but not duration. In conclusion, ‘excessive milk production’ during breast-feeding was associated with higher risk for early events independent of tumor characteristics, stressing the need to consider host factors in the evaluation of prognostic markers.

**Electronic supplementary material:**

The online version of this article (doi:10.1186/2193-1801-2-298) contains supplementary material, which is available to authorized users.

## Introduction

Breast cancer is the most common type of cancer among women in Sweden with nearly 8000 cases reported every year (The National Board of Health and Welfare 2011a). Breast cancer specific mortality is approximately 1500 women per year in Sweden (The National Board of Health and Welfare 2011b). Risk factors that influence the incidence of breast cancer may also affect survival after diagnosis (Barnett et al. 2008). Increased understanding of how lifestyle factors affect prognosis and tumor characteristics may contribute to better treatment.

Data from >50 000 breast cancer cases and >95 000 controls reveal that each year of breast-feeding decreases the risk by 4.3% (Collaborative Group on Hormonal Factors in Breast Cancer 2002), while others reported no overall association between breast-feeding and breast cancer (Michels et al. 1996). In women with *BRCA1* mutations, one year of breast-feeding was associated with between 32% and 45% decrease in the risk (Jernström et al. 2004; Kotsopoulos et al. 2012). Shorter breast-feeding duration has been associated with several factors such as *BRCA1* mutations (Jernström et al. 1998), body mass index (BMI) >30 (Guelinckx et al. 2011), and smoking (Hietala et al. 2008; Donath & Amir 2004). Low milk production has been associated with shorter duration of breast-feeding (Hietala et al. 2008; Gatti 2008). Whether insufficient milk production is associated with breast cancer risk is still unclear according to a review (Cohen et al. 2009). A murine model has suggested a link between breast tumor cells and insufficient maternal milk supply (Cohen et al. 2009; Lazar et al. 2000). The protective effect of breast-feeding, in high-risk *BRCA1* mutation carriers as well as women in general, is thought to be mediated in part by the decrease in post-lactational prolactin levels (Hietala et al. 2008), as well as a decrease in free testosterone levels (Nagata et al. 2011). One study showed that post-lactational prolactin levels were determined by breast-feeding duration of the first child and not simply by the first full-term pregnancy (Hietala et al. 2008). Additional pregnancies and prolonged total breast-feeding duration did not seem to further lower the hormonal levels (Hietala et al. 2008; Nagata et al. 2011). Women who reported insufficient milk production had higher prolactin levels (Hietala et al. 2008). The effect of milk production per se as opposed to breast-feeding duration on breast cancer is unknown. Prolactin increases the risk of breast cancer (Tworoger et al. 2007) and can promote cell proliferation and survival, increase cell motility, and support tumor vascularization (Tworoger & Hankinson 2006). However, in breast cancer cells, prolactin has also been implicated as an invasion suppressor hormone (Nouhi et al. 2006). According to two previous studies, there was no association between total breast-feeding duration and breast cancer survival (Trivers et al. 2007; Alsaker et al. 2011). To our knowledge, the effect of milk production on breast cancer-free survival has not been previously studied.

The breast is a dynamic tissue that develops throughout pregnancy, childbirth and lactation (Geddes 2007). Breast-feeding affects the lobulo-alveolar breast tissue and is associated with fewer type 1 lobules, which are rich in ER+ receptors (Russo et al. 2000). ER+ tumors grow more slowly and relapse later than ER– tumors (Osborne et al. 1980). The association between milk production and tumor characteristics or prognosis of women who subsequently develop breast cancer are unclear. Some studies have addressed the association between tumor characteristics and breast-feeding duration. Three studies reported that long breast-feeding was rare in patients with ER-/PgR- breast cancer (Palmer et al. 2011), or with triple negative breast cancer (Redondo et al. 2012; Gaudet et al. 2011). Others reported that longer breast-feeding was associated with reduced risk of basal-like breast cancer (Millikan et al. 2008), or that breast-feeding was a protective factor for luminal subtype when compared to non-luminal breast cancer (Turkoz et al. 2013).

The aims of the study were to investigate whether breast-feeding duration and milk production were associated with the tumor characteristics and prognosis of women who subsequently developed breast cancer.

## Material and methods

Between October 2002 and October 2008, women diagnosed with a first breast cancer at Skåne University Hospital in Lund, Sweden were invited to participate in an ongoing prospective study that focuses on how lifestyle factors influence prognosis and treatment response. Patients that had been treated for another cancer during the last 10 years were excluded. The study was approved by the Lund University Ethics Committee (Dnr LU75-02 & LU37-08). All participating patients signed informed consents. Six hundred and thirty four patients were included in the study during this period. Forty-two were excluded because of pre-operative treatment that may have affected tumor characteristics, leaving 592 pre-operatively untreated women. The follow-up rates of the patients without preoperative treatment who were alive and breast cancer-free were as follows for the 9, 7, 5, and 3-year: 80.0, 86.3, 92.6, and 93.5 %, respectively. Another 89 women were nulliparous and were excluded from the analyses regarding breast-feeding and milk production.

Patients received a pre-operative questionnaire on lifestyle and reproductive factors. The question regarding the duration of breast-feeding of each child was open. When the patient responded with an interval (e.g. 3–5 months), the highest value was used. In case of twins, only breast-feeding duration for one child was used. In regards to milk production for each child, the women were asked to pick between four alternatives: ‘almost no milk’, ‘insufficient’, ‘adequate’ or ‘excessive’. There was no question as to why a woman stopped breast-feeding. The question about smoking habits enquired whether the patients were currently smoking regularly, occasionally at parties, or not at all. A trained research nurse took body measurements, including weight, height, breast volume, and hip- and waist circumference. The nurse measured the volume of each breast, as previously described (Ringberg et al. 2006). After 3–6 months, 1, 2, 3, 5, 7, and 9 years, the patients received follow-up questionnaires.

Tumor characteristics were retrieved from pathology reports and included invasive tumor size, number of axillary lymph nodes involved, distant metastases and histologic grade. The expression of hormone receptors ER and PgR were analyzed. Tumors with >10% positive nuclear staining, were considered receptor positive (Bågeman et al. 2008; Jernström et al. 2009). Survival status and date of death were collected from the Swedish Population Registry. Clinical data including breast cancer events were obtained from patients’ charts.

### Statistics

SPSS Statistics 19 (IBM, Chicago, IL, USA) was used to analyze the data. Certain factors were not normally distributed. Year of birth was divided into three intervals, 1903–1940, 1941–1954 and 1955+, due to differing patterns of breast-feeding over time (The National Board of Health and Welfare 2010). Age at diagnosis was dichotomized, younger than 50 years versus 50 years and older and was also used as a proxy variable for menopause. It was not possible determine the exact age of menopause in patients who reported previous hormone therapy use and had hormone induced bleedings as well as in patients who reported hysterectomies without oophorectomies.

Breast-feeding duration of the first child was dichotomized into two groups, with the cut-point ≤12 months. Total breast-feeding duration for all children was also dichotomized, with the cut-point ≤12 months. Milk production was analyzed as a categorical variable including ‘almost no milk’, ‘insufficient, ‘adequate’ or ‘excessive’. The Chi-square test was used to analyze breast-feeding and milk production in relation to categorized variables. Reproductive factors included parity, age at first birth, breast-feeding duration, and amount of milk production for each child. Parity was classified as; 0, 1, 2, ≥3 children. Age at first birth was divided into four groups; <20, 20–24, 25–29, and ≥30 years.

BMI was stratified according to <25 kg/m^2^ (yes/no). Waist Hip Ratio (WHR) was defined as ≤0.85 (yes/no) (World Health Organization 2006; World Health Organization 2008). Breast volume was dichotomized as <850 ml or ≥850 ml (Markkula et al. 2012). Current smoking was defined as ‘yes’ or ‘no’. Women who reported smoking occasionally at parties were classified as smokers.

Disease-free survival was defined as absence of breast cancer events until the day of the last study follow-up or death, before January 1^st^, 2012. An event was classified as the occurrence of new breast cancer, local recurrence, regional or distant metastases. Patients with carcinoma *in situ* (n = 14) or a breast cancer event before three months after inclusion in study (n = 2) were excluded from the survival analyses. After excluding these patients and nulliparous patients, there were 64 breast cancer events including 39 distant metastases.

Survival in relation to breast-feeding duration >12 months (yes/no) and milk production for the first child ‘excessive’ (yes/no) was analyzed using Kaplan-Meier Log-Rank tests. Adjusted Hazard Ratios (HR) were analyzed using Cox regression. Adjustments were made for invasive tumor size (>20mm or muscular or skin involvement), any axillary lymph node involvement, ER and PgR status (positive/negative), and age (continuous), age at first full-term pregnancy (continuous), time since last full-term pregnancy (continuous), current smoking (yes/no), BMI <25 kg/m^2^ (yes/no), type of surgery including any reoperation modified radical mastectomy (yes/no), and treatment; tamoxifen (yes/no), aromatase inhibitor (yes/no), chemotherapy (yes/no), radiation therapy (yes/no).

The *P*-values were two-tailed and considered significant when <0.05. Nominal *P*-values were presented without adjustment for multiple testing.

## Results

### Patient characteristics

Characteristics of the 592 patients, regarding breast-feeding duration and milk production, are presented in Tables 1 and 2. The median value for year of birth for all women was 1945 (range 1903 to 1977). The median age of diagnosis was 59.9 years (range 25.8 to 99.9). As presented in Table 1, a significantly higher median age at first birth was seen among the women who had breast-fed their first child >12 months (*P*=0.02), compared to women with shorter breast-feeding duration, 29 years versus 24 years, respectively. The group of patients with a total breast-feeding duration of >12 months had a significantly lower percent of current smokers compared to the group with a total breast-feeding duration of ≤12 months (*P*=0.004). The median time since first pregnancy was 35.6 (range 0.8 to 80.9) years and the median time since last pregnancy was 30.9 (range 0.8 to 70.9) years.

**Table 1:** **Patient characteristics in relation to self-reported breast-feeding duration**

Table 1	All patients	Nulliparous	Breastfeeding duration of first child ≤12 months	Breastfeeding duration of first child >12 months	Ptrend or Chi-square, P	Total breastfeeding duration ≤12 months	Total breastfeeding duration >12 months	Ptrend or Chi-square, P
	n=592	n=89	n=476	n=20		n=325	n=178	
	n (percent)	n (percent)	n (percent)	n (percent)		n (percent)	n (percent)	
Year of birth								
1903-1940	171 (28.9%)	24 (27%)	14 (29.8%)	4 (20.0%)		95 (29.2%)	52 (29.2%)	
1941-1954	286 (48.3%)	47 (52.8%)	224 (47.1%)	10 (50.0%)	0.31	162 (49.8%)	77 (43.3%)	0.31
1955+	135 (22.8%)	18 (20.2%)	110 (23.1%)	6 (30.0%)		68 (20.9%)	49 (27.5%)	
Age at diagnosis (years)								
<50	120 (20.3%)	17 (19.1%)	98 (20.6%)	4 (20.0%)	1df; 0.95	63 (19.4%)	40 (22.5%)	1df; 0.41
≥50	472 (79.7%)	72 (80.9%)	378 (79.4%)	16 (80.0%)		262 (80.6%)	138 (77.5%)	
Body Mass Index (BMI), (kg/m-2)								
<25	312 (53.0%)	51 (57.3%)	244 (51.6%)	13 (65.0%)		167 (51.5%)	94 (53.4%)	
≥25-<30	182 (30.9%)	22 (24.7%)	154 (32.6%)	4 (20.0%)	0.40	106 (32.7%)	54 (30.7%)	0.81
≥30	95 (16.1%)	16 (18.0%)	75 (15.9%)	3 (15.0%)		51 (15.7%)	28 (15.9%)	
Missing	3	0	3	0		0	2	
Waist-Hip-Ratio (WHR)								
≤0,85	340 (57.6%)	51 (58.0%)	274 (57.7%)	11 (55.0%)	1df;0.81	183 (56.5%)	106 (59.6%)	1df; 0.51
>0,85	250 (42.4%)	37 (42.0%)	201 (42.3%)	9 (45.0%)		141 (43.5%)	72 (40.4%)	
Missing	2	1	1	0		1	0	
Breast volume (ml)								
<850	219 (37.0%)	33 (37.1%)	176 (37.0%)	8 (40.0%)	1df; 0.86	120 (36.9%)	66 (37.1%)	1df; 0.42
≥850	292 (49.3%)	40 (45.0%)	239 (50.2%)	10 (50.0%)		153 (47.1%)	99 (55.6%)	
Missing*	81	16	61	2		52	13	
Current smoking								
Yes	130 (22%)	24 (27.0%)	101 (21.2%)	2 (10.0%)	1df; 0.23	81 (24.9%)	25 (14.0%)	1df; 0.004
No	462 (78%)	65 (73.0%)	375 (78.8%)	18 (90.0%)		244 (75.1%)	153 (86.0%)	
Parity								
0	89 (15.0%)	89 (100%)	0	0		0	0	
1	91 (15.4%)		84 (17.6%)	6 (30.0%)		85 (26.2%)	6 (3.4%)	
2	253 (42.7%)		240 (50.4%)	10 (50.0%)	0.12	186 (57.2%)	67 (37.6%)	<0.0001
3+	159 (26.9%)		152 (31.9%)	4 (20.0%)		54 (16.6%)	105 (59.0%)	
Age at first birth, years**								
<20	62 (12.4%)		59 (12.4%)	3 (10.0%)		43 (13.3%)	19 (10.8%)	
20-24	187 (37.4%)		179 (37.8%)	5 (25.0%)	0.03	130 (40.1%)	57 (32.4%)	0.08
25-29	175 (35.0%)		168 (35.4%)	5 (25.0%)		104 (32.1%)	71 (40.3%)	
30+	76 (15.2%)		68 (14.3%)	8 (40.0%)		47 (14.5%)	29 (16.5%)	
Missing	3		2	0		1	2	
Milk production of first child **								
Almost no milk	25 (5.2%)		24 (5.3%)	0		23 (7.5%)	2 (1.2%)	
Too little milk	129 (27.0%)		128 (28.3%)	0		106 (34.5%)	23 (13.5%)	
Adequate amount of milk	245 (51.3%)		229 (50.6%)	13 (65.0%)	0.001	137 (44.6%)	108 (63.2%)	<0.0001
Too much milk	79 (16.5%)		72 (15.9%)	7 (35.0%)		41 (13.4%)	38 (22.2%)	
Missing	25		23	0		18	7	

* Data missing or previous breast surgery.

** Among parous women.

**Table 2:** **Patient characteristics in relation to self-reported milk production of the first child**

Table 2	All patients	Nulliparous	Milk production of first child -Almost no milk	Milk production of first child -Insufficient	Milk production of first child -Adequate	Milk production of first child -Excessive	Ptrend
	n=592	n=89	n=25	n=129	n=245	n=79	
	n (percent)	n (percent)	n (percent)	n (percent)	n (percent)	n (percent)	
Year of birth							
1903-1940	171 (28.9%)	24 (27.0%)	7 (28.0%)	35 (27.1%)	76 (31.0%)	21 (26.6%)	0.16
1941-1954	286 (48.3%)	47 (52.8%)	16 (64.0%)	69 (53.5%)	109 (44.5%)	34 (43.0%)	
1955+	135 (22.8%)	18 (20.2%)	2 (8.0%)	25 (19.4%)	60 (24.5%)	24 (30.4%)	
Age at diagnosis (years)							
<50	120 (20.3%)	17 (19.1%)	1 (4.0%)	22 (17.1%)	52 (21.2%)	23 (29.1%)	0.004
≥50	472 (79.7%)	72 (80.9%)	24 (96.0%)	107 (82.9%)	193 (78.8%)	56 (70.9%)	
Body Mass Index (BMI), (kg/m-2)							
<25	312 (53.0%)	51 (57.3%)	8 (32.0%)	62 (48.4%)	138 (56.3%)	41 (53.2%)	0.074
≥25-<30	185 (30.9%)	22 (24.7%)	8 (32.0%)	51 (39.8%)	68 (27.8%)	25 (32.5%)	
≥30	95 (16.1%)	16 (18.0%)	9 (36.0%)	15 (11.7%)	39 (15.9%)	11 (14.3%)	
Missing	3	0	0	1	0	2	
Waist-Hip-Ratio (WHR)							
≤0,85	340 (57.6%)	51 (58.0%)	12 (48.0%)	75 (58.1%)	146 (59.8%)	43 (54.4%)	0.82
>0,85	250 (42.4%)	37 (42.0%)	13 (53.0%)	54 (41.9%)	98 (40.2%)	36 (45.6%)	
Missing	2	1	0	0	1	0	
Breast volume (ml)							
<850	219 (37.0%)	33 (37.1%)	6 (24.0%)	54 (41.9%)	97 (39.6%)	20 (25.3%)	0.19
≥850	292 (49.3%)	40 (44.9%)	15 (60.0%)	58 (45.0%)	116 (47.3%)	50 (63.3%)	
Missing*	81	16	4	17	32	9	
Current smoking							0.19
Yes	130 (22%)	24 (27.0%)	9 (36.0%)	27 (20.9%)	44 (18.0%)	16 (20.3%)	
No	462 (78%)	65 (73.0%)	16 (64.0%)	102 (79.1%)	201 (82.0%)	63 (79.7%)	
Missing	0	0	0	0	0	0	
Parity							
0	89 (15.0%)	89 (100%)	0	0	0	0	0.43
1	91 (15.4%)		3 (12.0%)	22 (17.1%)	47 (19.2%)	15 (19.0%)	
2	253 (42.7%)		14 (56.0%)	64 (49.6%)	125 (51.0%)	40 (50.6%)	
3+	159 (26.9%)		8 (32.0%)	43 (33.3%)	73 (29.8%)	24 (30.4%)	
Missing	0	0	0	0	0	0	
Age at first birth, years**							
<20	62 (12.4%)		5 (20.0%)	16 (12.4%)	26 (10.7%)	10 (12.7%)	0.09
20-24	187 (37.4%)		8 (32.0%)	59 (45.7%)	73 (30.0%)	35 (44.3%)	
25-29	175 (35.0%)		11 (44.0%)	44 (34.1%)	93 (38.3%)	23 (29.1%)	
30+	76 (15.2%)		1 (4.0%)	10 (7.8%)	51 (21.0%)	11 (13.9%)	
Missing	3		0	0	2	0	
Breastfeeding duration of first child**							
Breastfeeding duration of first child, ≤ 12 months	476 (96.0%)		24 (100%)	128 (100%)	229 (94.6%)	72 (91.1%)	0.001
Breastfeeding duration of first child, > 12 months	20 (4%)		0 (%)	0 (%)	13 (5.4%)	7 (8.9%)	
Missing	7		1	1	3	0	

* Data missing or previous breast surgery.

** Among parous women.

As presented in Table 2, a lower age at diagnosis was significantly associated with a greater milk production (*P*_trend=_0.004). Milk production was not associated with other patient characteristics. Both longer breast-feeding duration of the first child and longer total breast-feeding duration were significantly associated with greater milk production while breast-feeding the first child (*P*_trend_=0.001) and (*P*_trend_<0.0001), respectively.

### Tumor characteristics

Table 3 presents the tumor characteristics of the 592 patients, in relation to breast-feeding duration. Shorter breast-feeding duration of the first child was associated with a higher frequency of ER+/PgR+ tumors (*P*=0.02). There was also a significant association between PgR+ tumors and shorter breast-feeding duration of the first child (*P*=0.02). ER–/PgR– tumors were more common among women who had breast-fed their first child >12 months, but this was not significant (*P*=0.09). The number of axillary lymph nodes involved was non-significantly higher among patients who had breast-fed their first child >12 months (*P*_trend_=0.09). Milk production was not associated with tumor characteristics, Table 4.

**Table 3:** **Tumor characteristics in relation to self-reported breast-feeding duration**

Table 3	All patients	Nulliparous	Breastfeeding duration of first child ≤12 months	Breastfeeding duration of first child >12 months	Ptrend or Chi-square, P	Total breastfeeding duration ≤12 months	Total breastfeeding duration >12 months	Ptrend or Chi-square, P
	n=592	n=89	n=476	n=20		n=325	n=178	
	n (percent)	n (percent)	n (percent)	n (percent)		n (percent)	n (percent)	
pT								
0	14 (2.4%)	3 (3.4%)	10 (2.1%)	0		9 (2.8%)	2 (1.1%)	
1	424 (71.6%)	61 (68.5%)	347 (72.9%)	12 (60.0%)		237 (72.9%)	126 (70.8%)	
2	144 (24.3%)	23 (25.8%)	111 (23.3%)	8 (40.0%)	0.20	72 (22.2%)	49 (27.5%)	0.47
3	9 (1.5%)	2 (2.2%)	7 (1.5%)	0		6 (1.8%)	1 (0.6%)	
4	1 (0.2%)	0	1 (0.2%)	0		1 (0.3%)	0	
Number of axillary lymph nodes								
0	368 (62.4%)	61 (68.5%)	296 (62.3%)	9 (47.4%)		203 (62.7%)	104 (58.8%)	
1-3	167 (28.3%)	21 (23.6%)	135 (28.4%)	6 (31.6%)	0.09	95 (29.3%)	51 (28.8%)	0.18
4+	55 (9.3%)	7 (7.9%)	44 (9.3%)	4 (21.1%)		26 (8.0%)	22 (12.4%)	
Missing	2	0	1	1		1	1	
Histologic grade								
I	157 (26.6%)	22 (24.7%)	132 (27.8%)	3 (15.0%)		87 (26.9%)	48 (27.0%)	
II	308 (52.1%)	47 (52.8%)	248 (52.2%)	11 (55.0%)	0.15	170 (52.5%)	91 (51.5%)	0.86
III	126 (21.3%)	20 (22.5%)	95 (20.0%)	6 (30.0%)		67 (20.7%)	39 (21.9%)	
Missing	1	0	1	0		1	0	
Hormone receptor status								
ER+	502 (86.7%)	76 (88.4%)	407 (87.2%)	15 (75.0%)	1df; 0.12	273 (85.8%)	153 (87.4%)	1df; 0.62
ER-	77 (13.3%)	10 (11.6%)	60 (12.8%)	5 (25.0%)		45 (14.2%)	22 (12.6%)	
PgR+	402 (69.4%)	61 (70.9%)	328 (70.2%)	9 (45.0%)	1df; 0.02	219 (68.9%)	122 (69.7%)	1df; 0.85
PgR-	177 (30.6%)	25 (29.1)	139 (29.8%)	11 (55.0%)		99 (31.1%)	53 (30.3%)	
Missing	13	3	9	0		7	3	
ER+ PgR+	398 (68.7%)	60 (69.8%)	325 (69.6%)	9 (45.0%)	1df; 0.02	216 (67.9%)	122 (69.7%)	1df; 0.68
ER+ PgR-	104 (18.0%)	16 (18.6%)	82 (17.6%)	6 (30.0%)	1df; 0.16	57 (17.9%)	31 (17.7%)	1df; 0.95
ER- PgR+	4 (0.7%)	1 (1.2%)	3 (0.6%)	0	1df; 0.72	3 (0.9%)	0	1df; 0.20
ER- PgR-	73 (12.6%)	9 (10.5%)	57 (12.2%)	5 (25.0%)	1df; 0.09	42 (13.2%)	22 (12.6%)	1df; 0.84
Missing	13	3	9	0		7	3

**Table 4:** **Tumor characteristics in relation to self-reported milk production**

Table 4	All patients	Nulliparous	Milk production of first child -Almost no milk	Milk production of first child -Insufficient	Milk production of first child -Adequate	Milk production of first child -Excessive	Ptrend
	n=592	n=89	n=25	n=129	n=245	n=79	
	n (percent)	n (percent)	n (percent)	n (percent)	n (percent)	n (percent)	
pT							0.79
0	14 (2.4%)	3 (3.4%)	2 (8.0%)	2 (1.6%)	3 (1.2%)	1 (1.3%)	
1	424 (71.6%)	61 (68.5%)	15 (60.0%)	89 (69.0%)	185 (75.5%)	57 (72.2%)	
2	144 (24.3%)	23 (25.8%)	8 (32.0%)	36 (27.9%)	54 (22.0%)	19 (24.1%)	
3	9 (1.5%)	2 (2.2%)	0	2 (1.6%)	3 (1.2%)	2 (2.5%)	
4	1 (0.2%)	0	0	0	0	0	
Number of axillary lymph nodes							0.14
0	368 (62.4%)	61 (68.5%)	18 (72.0%)	81 (62.8%)	151 (61.9%)	43 (55.1%)	
1-3	167 (28.3%)	21 (23.6%)	4 (16.0%)	38 (29.5%)	72 (29.5%)	24 (30.8%)	
4+	55 (9.3%)	7 (7.9%)	3 (12.0%)	10 (7.8%)	21 (8.6%)	11 (14.1%)	
Missing	2	0	0	0	1	1	
Histologic grade							0.29
I	157 (26.6%)	22 (24.7%)	11 (44.0%)	31 (24.2%)	66 (26.9%)	23 (29.1%)	
II	308 (52.1%)	47 (52.8%)	12 (48.0%)	73 (57.0%)	123 (50.2%)	39 (49.4%)	
III	126 (21.3%)	20 (22.5%)	2 (8.0%)	24 (18.8%)	56 (22.9%)'	17 (21.5%)	
Missing	1	0	0	1	0	0	
Hormone receptor status							
ER+	502 (86.7%)	76 (88.4%)	22 (95.7%)	108 (84.4%)	207 (85.9%)	71 (91.0%)	0.68
ER-	77 (13.3%)	10 (11.6%)	1 (4.3%)	20 (15.6%)	34 (14.1%)	7 (9.0%)	
PgR+	402 (69.4%)	61 (70.9%)	18 (78.3%)	85 (66.4%)	162 (67.2%)	58 (74.4%)	0.70
PgR-	177 (30.6%)	25 (29.1)	5 (21.7%)	43 (33.6%)	79 (32.8%)	20 (25.6%)	
Missing	13	3	2	1	4	1	
ER+ PgR+	398 (68.7%)	60 (69.8%)	18 (78.3%)	85 (66.4%)	159 (66.0%)	58 (74.4%)	0.76
ER+ PgR-	104 (18.0%)	16 (18.6%)	4 (17.4%)	23 (18.0%)	48 (19.9%)	13 (16.7%)	0.98
ER- PgR+	4 (0.7%)	1 (1.2%)	0	0	3 (1.2%)	0	0.65
ER- PgR-	73 (12.6%)	9 (10.5%)	1 (4.3%)	20 (15.6%)	31 (12.9%)	7 (9.0%)	0.60
Missing	13	3	2	1	4	1	

### Risk for early breast cancer events

**Figure 1:**
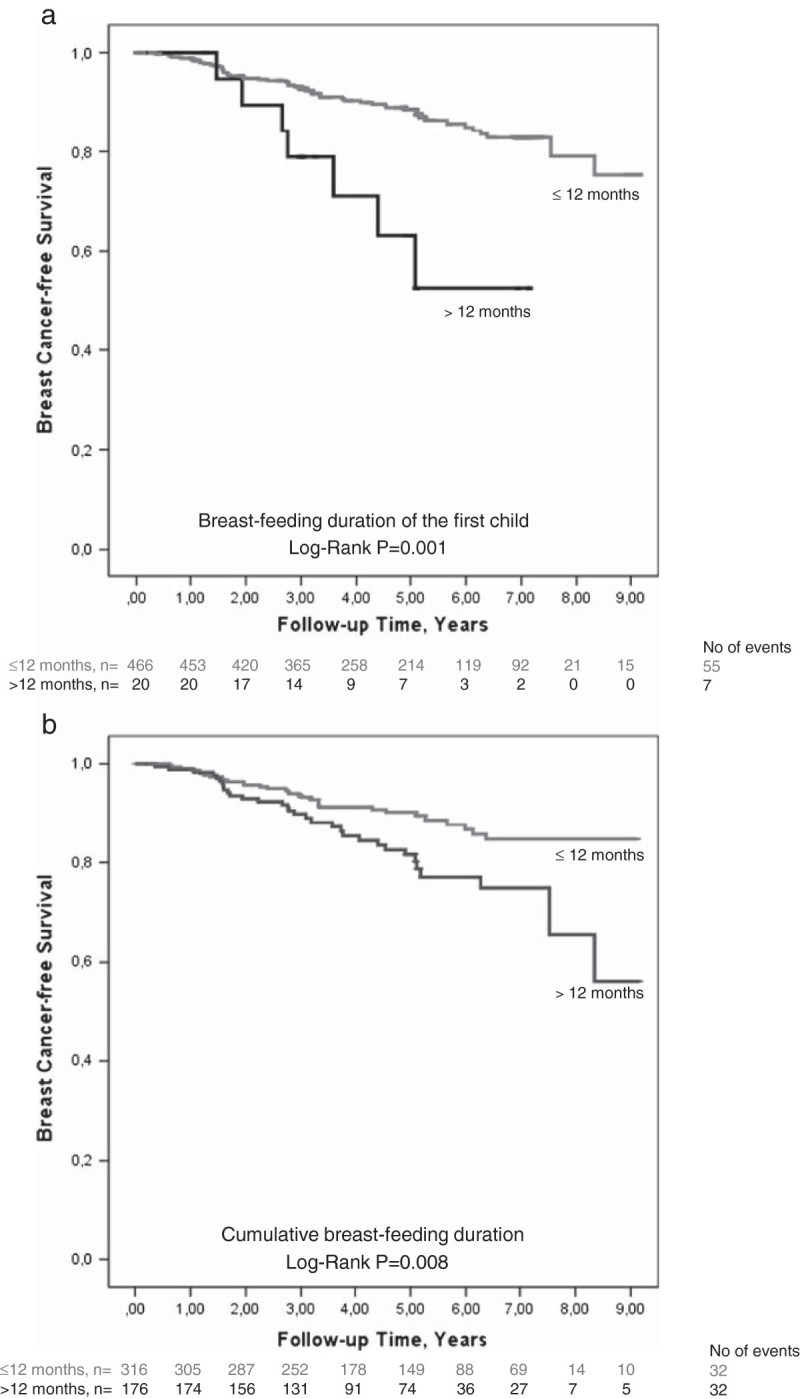
**Kaplan-Meier estimate of breast cancer-free survival in relation to breast-feeding duration. a**. Kaplan-Meier estimate of breast cancer-free survival in relation to breast-feeding duration of first child (*P*=0.001). The number of patients at each follow-up is indicated. Since this is an ongoing study, the number of patients decreases with each follow-up. **b**. Kaplan-Meier estimate of breast cancer-free survival in relation to total breast-feeding duration (*P*=0.008). The number of patients at each follow-up is indicated. Since this is an ongoing study, the number of patients decreases with each follow-up.

Risk for early breast cancer events was analyzed in relation to breast-feeding duration of the first child (Figure 1a) and total breast-feeding duration (Figure 1b). Median follow-up time was 4.9 (interquartile range 3.0 to 6.4) years for all patients. Higher risk for early events was seen in patients with over >12 months of breast-feeding of the first child (Log-Rank *P*=0.001) and over > 12 months of total breast-feeding (Log-Rank *P*=0.008). Breast-feeding of the first child > 12 months was associated with a three-fold increased risk for an early event crude HR 3.54 (95% CI: 1.61-7.79) and remained significant after adjustment for age, invasive tumor size (>20 mm or muscular or skin involvement), axillary lymph node involvement, ER and PgR status, age at first birth, time since last birth, smoking status, and BMI, type of surgery, and treatment, adjusted HR 2.90 (95% CI: 1.22-6.94). Total breast-feeding >12 months was associated with a two-fold increased risk for an early event crude HR 1.92 (1.18-3.14) and adjusted HR 1.96 (1.12-3.41).

**Figure 2:**
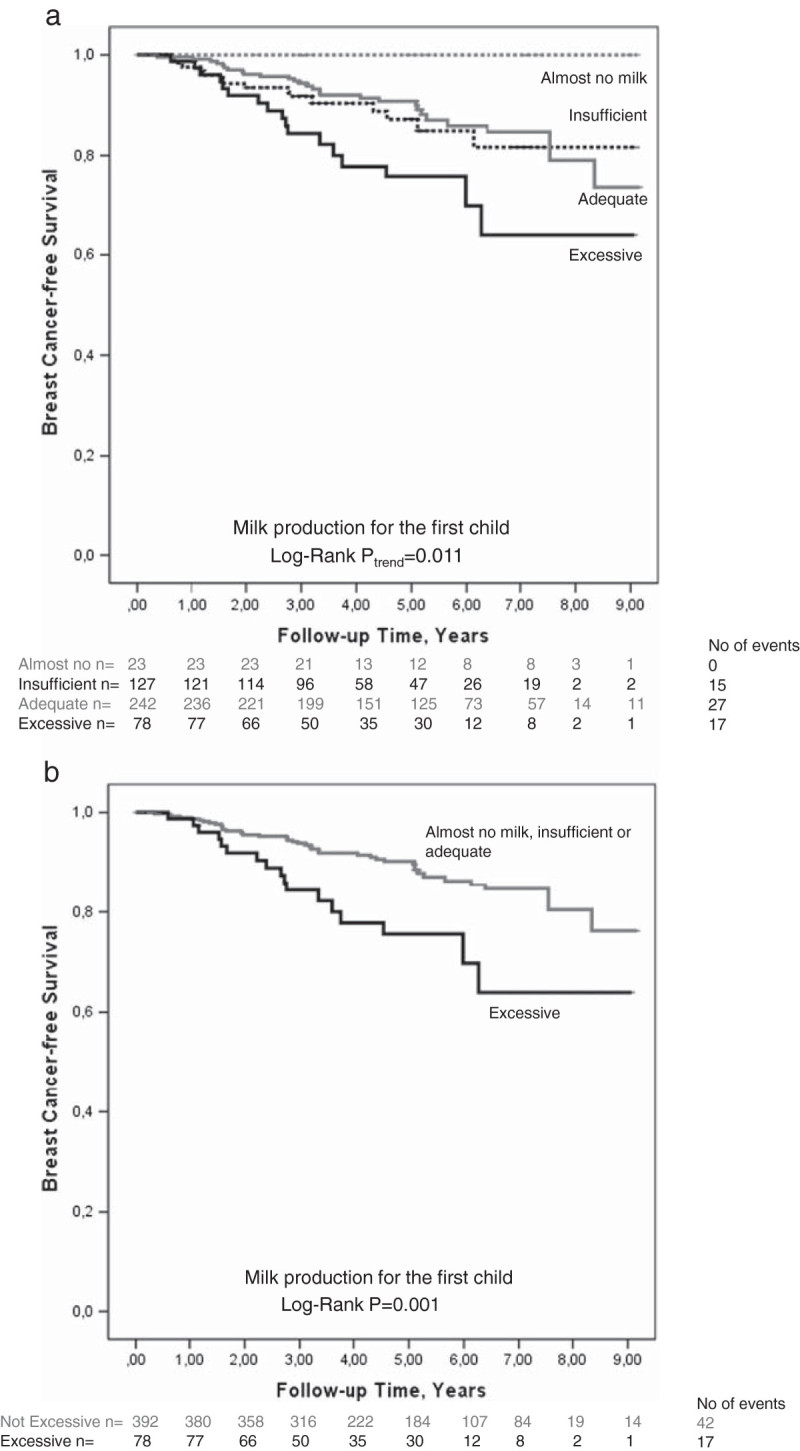
**Kaplan-Meier estimate of breast cancer-free survival in relation to milk production. a**. Kaplan-Meier estimate of breast cancer-free survival in relation to four categories of milk production (*P*=0.011). The number of patients at each follow-up is indicated. Since this is an ongoing study, the number of patients decreases with each follow-up. **b**. Kaplan-Meier estimate of breast cancer-free survival in relation to ‘excessive milk production’ (*P*=0.001). In this figure ‘excessive milk production’ is compared to the three categories with lower milk production. The number of patients at each follow-up is indicated. Since this is an ongoing study, the number of patients decreases with each follow-up.

Figures 2a and 2b illustrate the associations between milk production during breast-feeding of the first child and risk for early events. Greater milk production was associated with higher risk for early events (Figure 2a; Log-Rank *P*_trend_=0.011). Patients with almost no milk had no events. ‘Excessive milk production’ was associated with an over two-fold increased risk for early breast cancer events compared to patients with either adequate or insufficient milk production as presented in Figure 2b (Log-Rank *P*=0.001) crude HR 2.44 (1.39-4.30) and adjusted HR 2.54 (1.37-4.68). ‘Excessive milk production’ was associated with higher risk for early events both in patients <50 years and ≥ 50 years at diagnosis.

In a multivariable model including both ‘excessive milk production’ and breast-feeding duration of the first child >12 months, both were associated with a 2-fold risk for early events, adjusted HRs 2.33 (1.25-4.36) and 2.39 (0.97-5.85), respectively. There was no interaction between long breast-feeding duration of the first child and ’excessive milk production’, neither in the crude analysis nor in the multivariable model (both *P* s≥0.54). However, when including ‘excessive milk production’ and total breast-feeding duration >12 months in the same model, total breast-feeding duration was only borderline significantly associated with risk for early events (*P*=0.14), while the effect of milk production remained significant (*P*=0.007).

‘Excessive milk production’ and breast-feeding duration of the first child >12 months, were both associated with shorter distant metastasis-free survival, adjusted HRs 2.59 (1.13-5.94) and 2.44 (0.82-7.29), respectively. In the model including ‘excessive milk production’ and total breast-feeding duration >12 months in the same model showed that patients with ‘excessive’ milk production had an over two-fold increased risk for early distant metastases adjusted HR 2.88 (1.26-6.55), while total breast-feeding duration >12 months was not associated with distant-metastasis-free survival HR 1.32 (0.62-2.82).

## Discussion

The main findings of this study were that breast cancer patients who reported to have experienced ‘excessive milk production’ while breast-feeding had an over two-fold increased risk of an early breast cancer event as well as distant metastasis. On the other hand, none of the breast cancer patients in this study who had experienced ‘almost no milk production’ had a breast-cancer event. To our knowledge this has not been shown in previous studies. Longer breast-feeding duration of the first child was also associated with higher risk for early events. This finding could only in part be explained by the amount of milk produced. Breast-feeding duration was associated with ER/PgR status but not with other tumor characteristics and milk production was not associated with any of the tumor characteristics investigated.

Breast-feeding the first child for >12 months was associated with a higher median age at first birth. Different distributions of breast-feeding duration in relation to patient and tumor characteristics were also originally investigated. However, none of these distributions contributed any further to the study and it was decided to make the cut-off at 1-year, which is in line with other studies (Collaborative Group on Hormonal Factors in Breast Cancer 2002; Jernström et al. 2004). Ludvigsson *et al.* reported that younger women tended to breast-feed for a shorter period of time (Ludvigsson & Ludvigsson 2005). Women who had a total breast-feeding duration of >12 months were significantly less often current smokers in the present study, which is in line with other studies (Donath & Amir 2004; Ludvigsson & Ludvigsson 2005). However, our study only considered current smoking. Current smoking was not significantly associated with milk production of the first child, which was significantly correlated to breast-feeding duration of the first child, as well as total breast-feeding duration. Accordingly, women with ‘excessive milk production’ tended to breast-feed for a longer period of time, which was expected, since an insufficient amount of milk makes prolonged breast-feeding more difficult (Gatti 2008; Thulier & Mercer 2009). Shorter breast-feeding is associated with several socio-economic factors including low maternal education (Ludvigsson & Ludvigsson 2005). In the current study there was no question regarding socioeconomic status. Low socioeconomic status is associated with a decreased risk for breast cancer (Granstrom et al. 2008), but an increased risk for recurrence and death (Halmin et al. 2008). Socioeconomic status is therefore unlikely to explain why patients who reported longer breast-feeding duration and ‘excessive milk production’ had increased risk of early events in the current study.

Breast-feeding duration, but not milk production, was associated with ER and PgR status but not with other tumor characteristics. In the current study, ER–/PgR– tumors were non-significantly more common among women who had breast-fed their first child for >12 months. However, there were only 20 women who had breast-fed their first child for >12 months. Conversely, two studies have found that triple negative breast tumors were rare in patients with longer breast-feeding duration (Redondo et al. 2012; Gaudet et al. 2011). However, the study by Gaudet *et al.* only analyzed women 56 years and younger, while the current study included patients of all ages. Since Her2-status has only been routinely evaluated since November 2005 in Lund (Lundin et al. 2011), it was not possible to investigate triple negativity in relation to breast-feeding duration or milk production in the current study.

Longer breast-feeding duration, both total and of the first child, was associated with a higher risk for early events. ‘Excessive milk production’ was the strongest factor of breast-feeding, affecting the risk for early events as it was significant whether the model included breast-feeding of the first child or total breast-feeding duration. None of the investigated tumor criteria were associated with milk production. However, it is possible that PRL receptor or androgen receptor (AR) expression may differ as breast-feeding affects both prolactin and testosterone levels (Hietala et al. 2008; Nagata et al. 2011). Ormandy *et al.* reported that the level of PRL receptor expression in breast cancer cell lines was linearly related to that of the ER and PgR expression, but not to that of the AR (Ormandy et al. 1997).

A cleaved fragment of prolactin called PRL 16K, which is generated during breast-feeding, has been shown to inhibit angiogenesis (Freeman et al. 2000; Goffin et al. 1999). Inhibited angiogenesis may result in more invasive tumors and more metastases according to a recent review (Leite de Oliveira et al. 2011). Longer breast-feeding duration may thereby affect tumor characteristics through impaired angiogenesis. However, no increased axillary lymph node involvement was found among tumors from patients with ‘excessive milk production’. Angiogenesis in breast tumors is not routinely analyzed in Sweden today.

We did not find any significant association between milk production and tumor characteristics. However, the finding of an increased risk of early breast cancer events among the women with ‘excessive milk production’ may indicate tumors with a higher proliferation rate. Further analyzes, including milk production, PRL levels, AR expression, and proliferation rate (Ki67), need to be done to verify this theory. Ki67 expression has been routinely analyzed in Lund since March 2009 (Lundin et al. 2011).

Breast-feeding duration was not as strongly associated with early events as we hypothesized. A recent larger cohort study from Norway reported that there was no evidence for a dose-related effect of breast-feeding on survival (Alsaker et al. 2011), but this study did not specifically look at breast-feeding duration of the first child. Similar results were found in another smaller Swedish retrospective study that only included long-term survivors (Lööf-Johanson et al. 2011).

Between October 2002 and October 2008, 1139 women were registered with breast cancer at Skåne University Hospital in Lund, according to the Regional Tumor Registry. In this study, 592 patients were included. Most of the remaining women did not decline participation in the study. Instead it was the lack of available research nurses that limited the number of participants. The women who did not participate in this study had similar patient and tumor characteristics as the ones who did participate (Lundin et al. 2011). Therefore, we consider the material to be generalizable for the women operated for breast cancer in Lund.

In Sweden, a very high proportion of women initiate breast-feeding and almost all (97%) infants born in 2008 were breastfed at the age of one week (The National Board of Health and Welfare 2010). This means that our study results may be generalizable to the rest of Sweden, but may not be generalizable to countries with different breast-feeding practices. Since our study was a prospective cohort, we have been able to minimize several types of bias. A retrospective study would not have caught the short-term survivors, and therefore would have favored patients with less aggressive cancer. All patients were included in the study in a similar way; a few days prior to surgery and before any events, minimizing the risk for recall bias.

A study limitation is the fact that breast-feeding duration and milk production were self-reported and that there was no question with respect to why a woman stopped breast-feeding. For some of the women, many years had passed since they gave birth and there is a risk that they could have forgotten for how long they breast-fed and how much milk they produced. However, giving birth and breast-feeding are very important events in a woman’s life. In Sweden, new mothers who have too much milk are encouraged, during postnatal follow-up, to sell their extra breast milk to the hospital to feed premature babies, as long as the woman is healthy and a non-smoker. Objective data on milk production would be hard to come by given that it would involve measuring breast milk production in a large cohort of healthy women several decades prior to breast cancer diagnosis. Inaccurate reporting of breast-feeding duration and milk production would have biased the results towards the null hypothesis.

Milk production during breast-feeding may merit further study to evaluate if it can be used as a prognostic factor, since no correlation between milk production and tumor characteristics was observed. Asking about breast-feeding and milk production history is cheaper. Markers, which would help to better tailor adjuvant therapy to each patient are urgently needed. If the association between ‘excessive milk production’ and increased risk for early events is confirmed in other breast cancer populations, patients with ‘excessive milk production’ may warrant more aggressive breast cancer therapy and/or closer follow-up while patients who report almost no milk production while breast-feeding their first child may warrant less aggressive treatment with fewer side-effects. However, the median follow-up is currently only five years and ER positive tumors tend to relapse late (Osborne et al. 1980). Since breast-feeding is protective with respect to breast cancer incidence (Collaborative Group on Hormonal Factors in Breast Cancer 2002), we hypothesize that women who develop breast cancer in spite of long breast-feeding may get especially aggressive cancers, as shown in another South Swedish cohort (Butt et al. 2010).

In conclusion, our finding that production of an ‘excessive’ amount of milk during breast-feeding increases the risk for early event highlights the potential importance of milk production as a reproductive factor that may affect breast cancer prognosis. This could potentially lead to an adjusted way of asking about patient history, regarding reproductive factors for breast cancer patients. Patients with an excessive milk production seem to have a more aggressive type of breast cancer, with higher risk for early breast cancer events. Therefore they may need a different treatment than patients with almost no milk production, among whom no early events were observed.

## Authors’ original submitted files for images

Below are the links to the authors’ original submitted files for images. Authors’ original file for figure 1 Authors’ original file for figure 2 Authors’ original file for figure 3 Authors’ original file for figure 4
