# Supplementary figures and images for: Excessive milk production during breast-feeding prior to breast cancer diagnosis is associated with increased risk for early events
Source: Springerplus. 2013 Jul 3;2(1):298. doi: 10.1186/2193-1801-2-298 (PMC3706724; doi:10.1186/2193-1801-2-298)

a

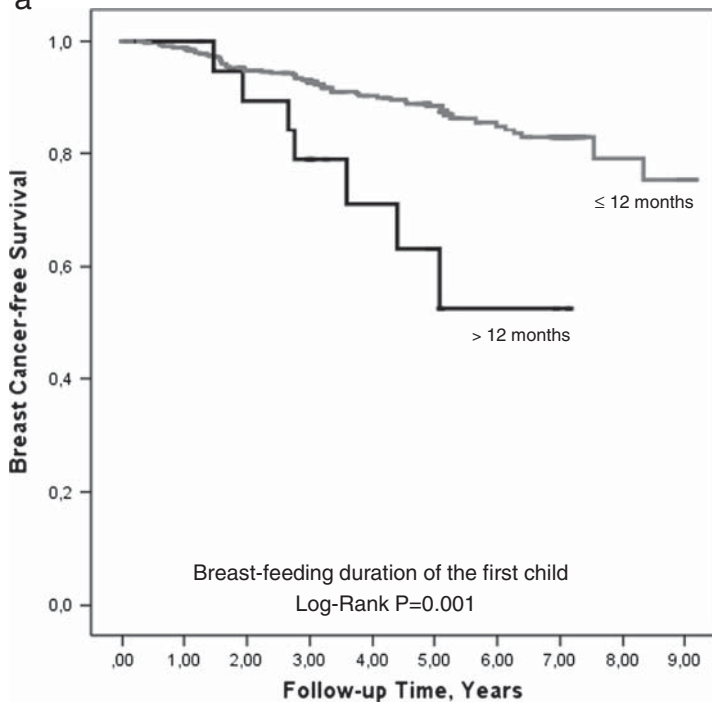

|                    | 1   | 2   | 3   | 4   | 5   | 6   | 7  | 8  | 9  | 10 | No of events |
|--------------------|-----|-----|-----|-----|-----|-----|----|----|----|----|--------------|
| ≤12 months, n= 466 | 453 | 420 | 365 | 258 | 214 | 119 | 92 | 21 | 15 |    | 55           |
| >12 months, n= 20  | 20  | 17  | 14  | 9   | 7   | 3   | 2  | 0  | 0  |    | 7            |

b

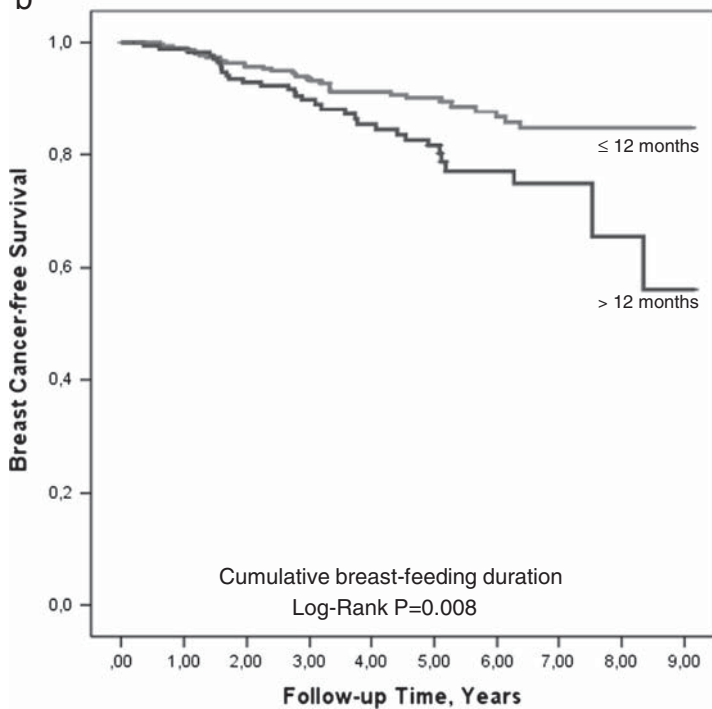

| Follow-up Time, Years |     |     |     |     |     |     |    |    |    | No of events |    |
|-----------------------|-----|-----|-----|-----|-----|-----|----|----|----|--------------|----|
| ≤12 months, n=        | 316 | 305 | 287 | 252 | 178 | 149 | 88 | 69 | 14 | 10           | 32 |
| >12 months, n=        | 176 | 174 | 156 | 131 | 91  | 74  | 36 | 27 | 7  | 5            | 32 |

Supplement: Supplementary file 1 — Authors’ original file for figure 1 [file 40064_2013_362_MOESM1_ESM.pdf]
