# Supplementary material for: Excessive milk production during breast-feeding prior to breast cancer diagnosis is associated with increased risk for early events
Source: Springerplus. 2013 Jul 3;2(1):298. doi: 10.1186/2193-1801-2-298 (PMC3706724; doi:10.1186/2193-1801-2-298)
Supplement: Supplementary file 2 — Authors’ original file for figure 2 [file 40064_2013_362_MOESM2_ESM.pdf]

a

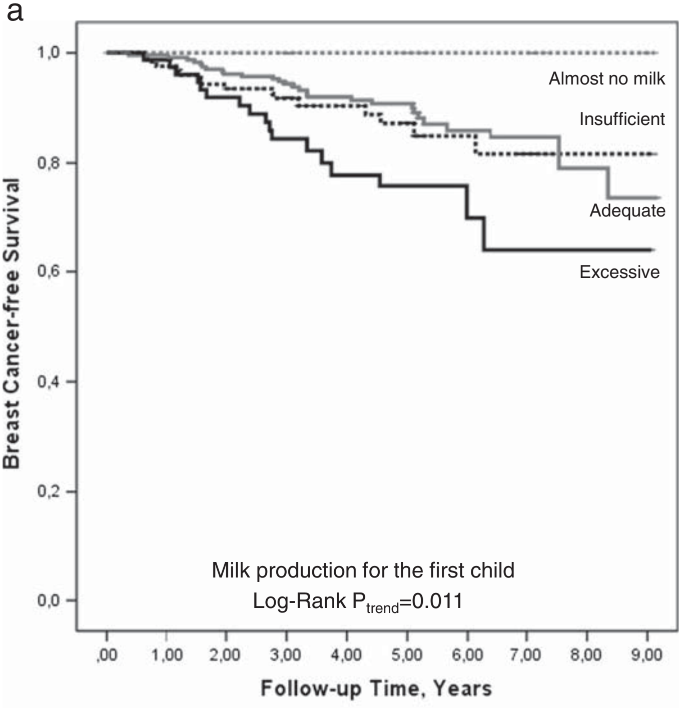

											No of events
Almost no n=	23	23	23	21	13	12	8	8	3	1	0
Insufficient n=	127	121	114	96	58	47	26	19	2	2	15
Adequate n=	242	236	221	199	151	125	73	57	14	11	27
Excessive n=	78	77	66	50	35	30	12	8	2	1	17

b

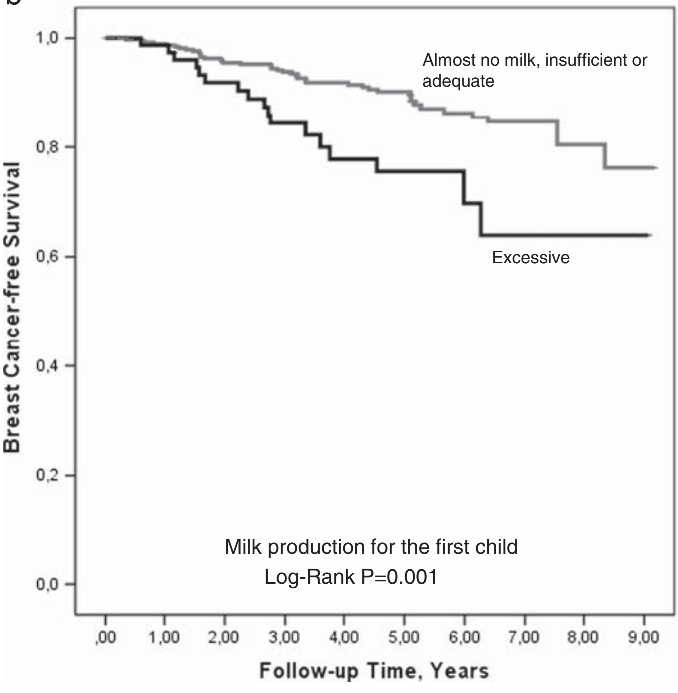

											No of events
Not Excessive n=	392	380	358	316	222	184	107	84	19	14	42
Excessive n=	78	77	66	50	35	30	12	8	2	1	17
