# Supplementary material for: Excessive milk production during breast-feeding prior to breast cancer diagnosis is associated with increased risk for early events
Source: Springerplus. 2013 Jul 3;2(1):298. doi: 10.1186/2193-1801-2-298 (PMC3706724; doi:10.1186/2193-1801-2-298)
Supplement: Supplementary file 3 — Authors’ original file for figure 3 [file 40064_2013_362_MOESM3_ESM.ppt]

## Slide 1
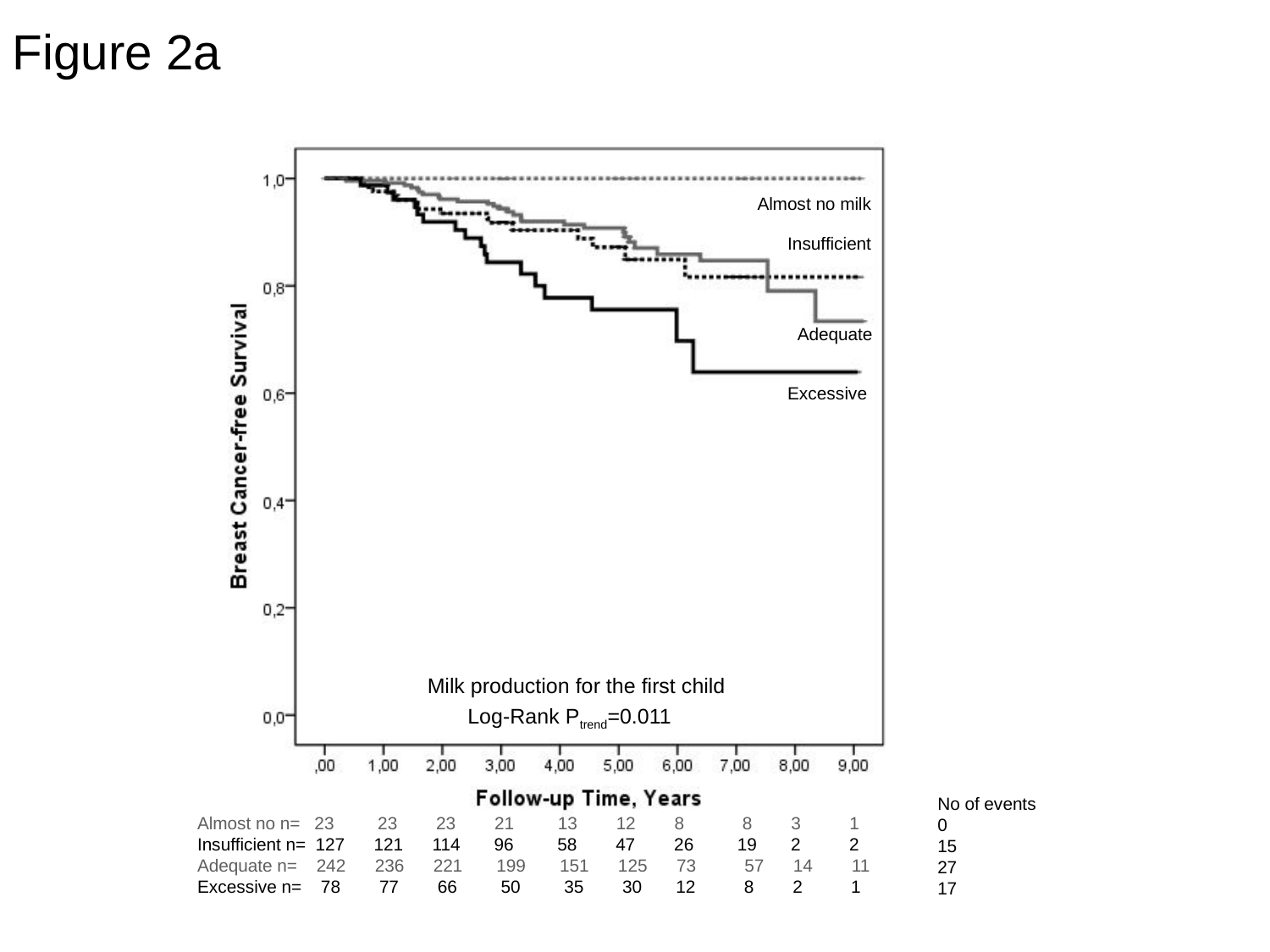

Figure 2a
Almost no milk
Insufficient
Adequate
Excessive
Milk production for the first child
Log-Rank Ptrend=0.011
No of events
0
15
27
17
Almost no n= 23 23 23 21 13 12 8 8 3 1
Insufficient n= 127 121 114 96 58 47 26 19 2 2
Adequate n= 242 236 221 199 151 125 73 57 14 11
Excessive n= 78 77 66 50 35 30 12 8 2 1
