# Supplementary material for: Excessive milk production during breast-feeding prior to breast cancer diagnosis is associated with increased risk for early events
Source: Springerplus. 2013 Jul 3;2(1):298. doi: 10.1186/2193-1801-2-298 (PMC3706724; doi:10.1186/2193-1801-2-298)
Supplement: Supplementary file 4 — Authors’ original file for figure 4 [file 40064_2013_362_MOESM4_ESM.ppt]

## Slide 1
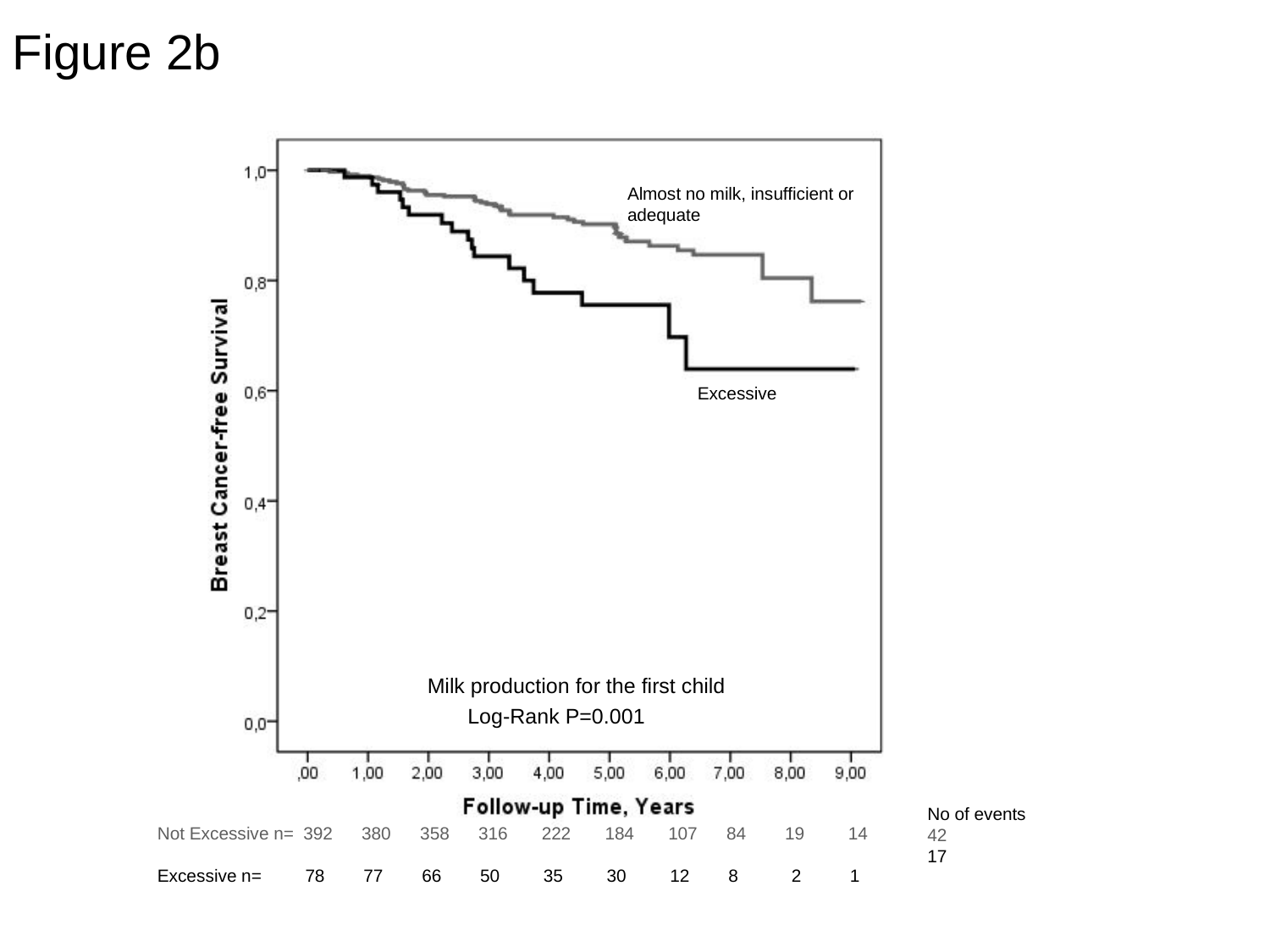

Figure 2b
Almost no milk, insufficient or adequate
Excessive
Milk production for the first child
Log-Rank P=0.001
No of events
42
17
Not Excessive n= 392 380 358 316 222 184 107 84 19 14
Excessive n= 78 77 66 50 35 30 12 8 2 1
